# Supplementary material for: VenoMS—A Website for the Low Molecular Mass Compounds in Spider Venoms
Source: Metabolites. 2020 Aug 11;10(8):327. doi: 10.3390/metabo10080327 (PMC7464580; doi:10.3390/metabo10080327)
Supplement: Supplementary file 1 [file metabolites-10-00327-s001.zip › metabolites-805127-sup.pdf]

Supplementary material for:

venoMS – A Database for the Low Molecular Mass Compounds in Spider Venoms

Yvonne M. Forster [1], Silvan Reusser [2], Florian Forster [2], Stefan Bienz [1] and Laurent Bigler [1,*]

[1] University of Zurich, Department of Chemistry, Winterthurerstrasse 190, 8057 Zurich, Switzerland
[2] CAOS AG, Teufener Strasse 19, 9000 St. Gallen, Switzerland
* Correspondence: laurent.bigler@chem.uzh.ch; Tel.: +41-44-635-68-12

Supplementary Figure 1: UHPLC-HR-ESI-MS(/MS) data acquired with the SAM2020 method and used for the characterization of IndAc4333 from the venom of *A. aperta*, and the comparison of this data with the values generated by FRIOC (full scan values before and after H/D exchange and MS/MS fragment ion masses).

Supplementary Figure 2: : UHPLC-HR-ESI-MS(/MS) data acquired with the SAM2020 method and used for the characterization of 4-OH-IndAc3(OH)335(NMe_3)$^+$ from the venom of *A. aperta*, and the comparison of this data with the values generated by FRIOC (full scan values before and after H/D exchange and MS/MS fragment ion masses).

Supplementary Figure 3: UHPLC-HR-ESI-MS(/MS) data acquired with the SAM2020 method and used for the characterization of 4-OH-IndAc3(OH)335(NMe_2) from the venom of *A. aperta*, and the comparison of this data with the values generated by FRIOC (full scan values before and after H/D exchange and MS/MS fragment ion masses).

Supplementary Figure 1: IndAc4333 (*Agelenopsis aperta*)

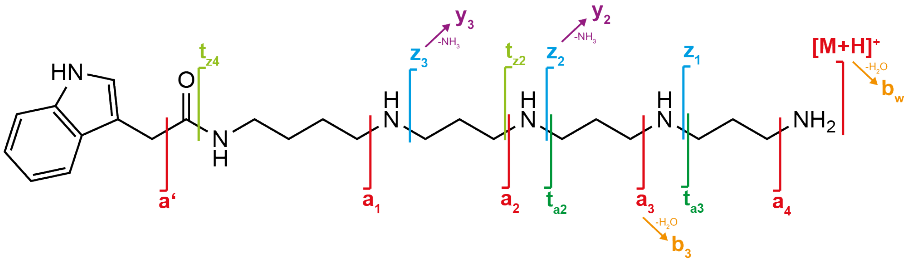

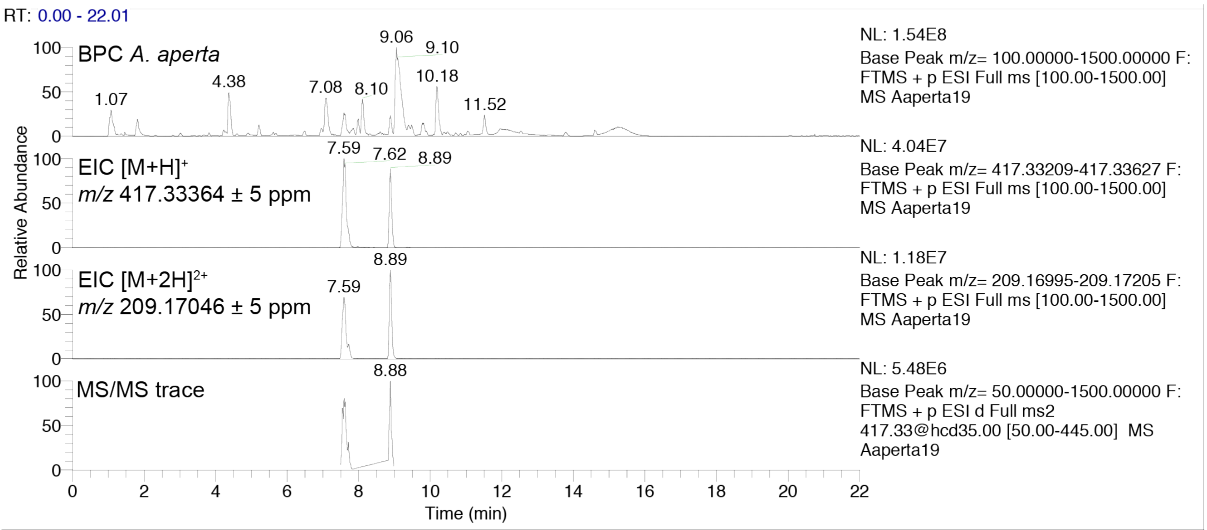

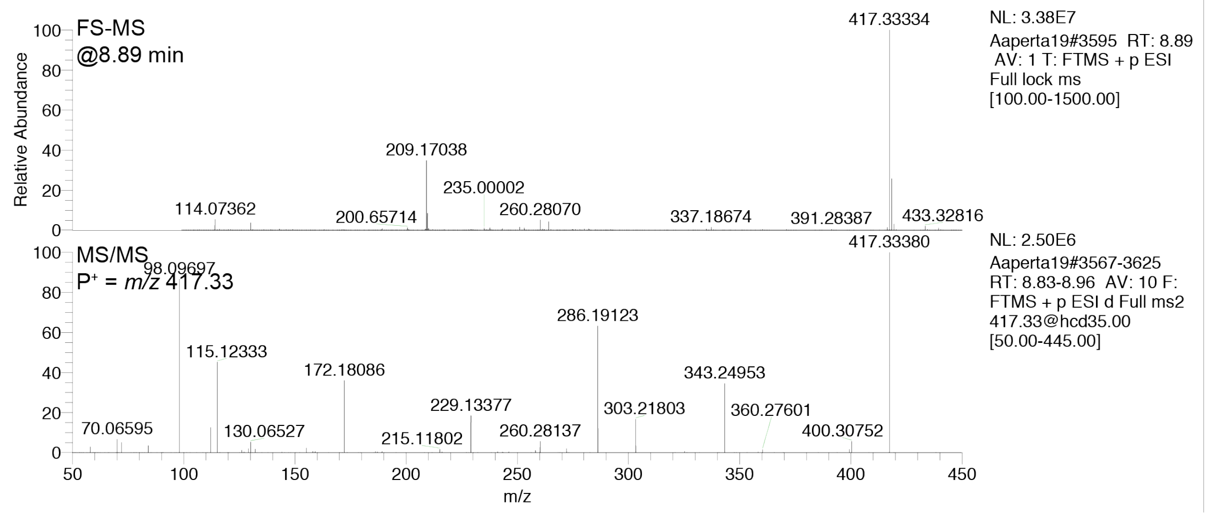

HDX
BPC *A. aperta*

HDX
EIC $[M(D_7)+D]^+$
m/z 425.38385 ± 5 ppm

HDX
EIC $[M(D_7)+2D]^{2+}$
m/z 213.69870 ± 5 ppm

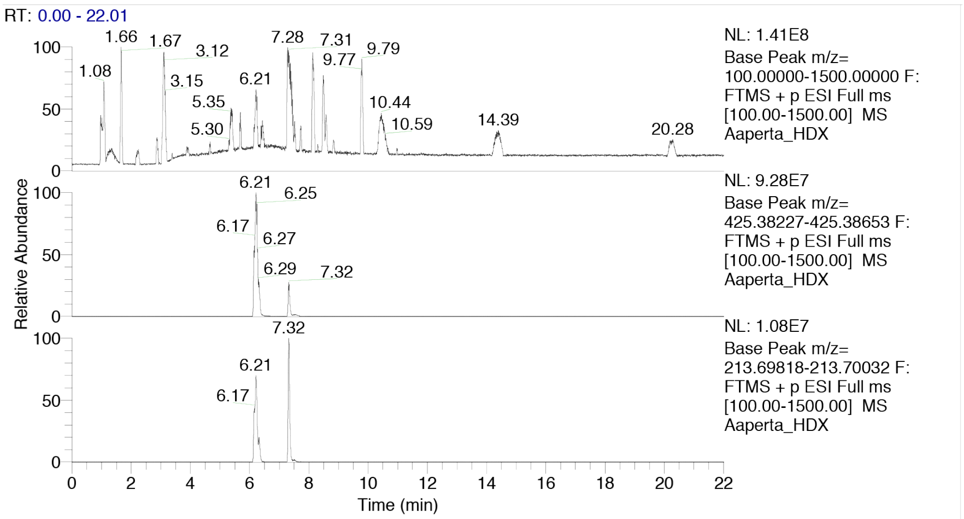

HDX
FS-MS
@ 7.32 min

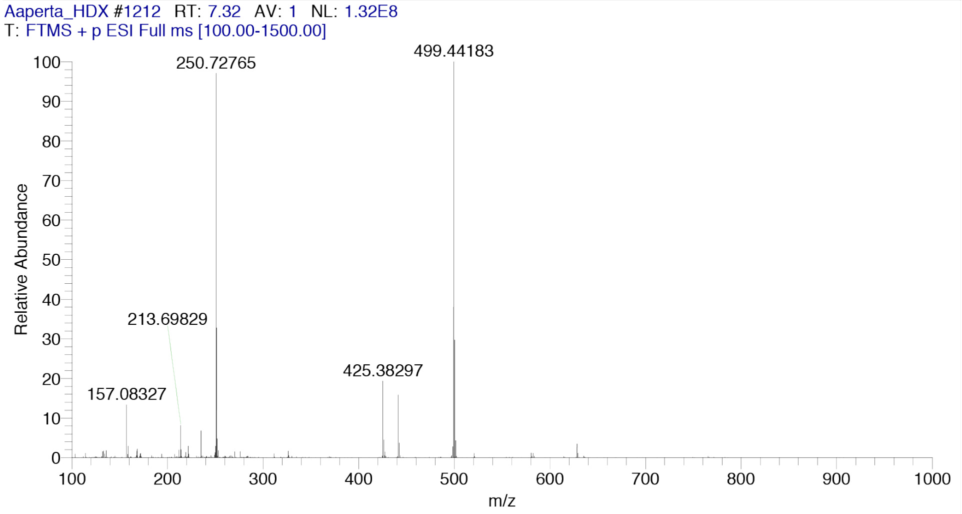

FS-MS / FS-MS HDX

	m/z detected	R_t [min]	*m/z* FRIOC	Molecular formula	Δ [ppm]
$[M+H]^+$	417.33334	8.89	417.33364	$C_{23}H_{41}N_6O^+$	-0.72
$[M(D_7)+D]^+$	425.38297	7.32	425.38385	$C_{23}H_{33}D_8N_6O^+$	-2.07

MS/MS fragment ions (P^+ = *m/z* 417.33)

m/z detected	Rel. Int.	*m/z* FRIOC	Molecular formula	Δ [ppm]	Fragment type
58.06602	2.82	58.06513	$C_3H_8N^+$	15.33	z_1
70.06595	6.68	–			
72.08155	5.08	–			
84.08158	3.45	–			
98.09697	86.36	98.09643	$C_6H_{12}N^+$	5.50	y_2
112.11246	12.67	–	$C_7H_{14}N^+$	3.39	y_2' *
115.12333	45.27	115.12297	$C_6H_{15}N_2^+$	3.13	z_2
126.12816	1.44	–			
129.13899	1.93	–	$C_7H_{17}N_2^+$	2.79	z_2' *
130.06528	5.26	–	$C_9H_8N^+$	1.15	a' *
132.14979	1.80	132.14952	$C_6H_{18}N_3^+$	2.04	tz_2
155.15458	2.25	155.15428	$C_9H_{19}N_2^+$	1.93	y_3
158.06061	0.61	–	$C_{10}H_8NO^+$	3.61	a_0 *
159.09208	0.50	–			
172.18086	36.00	172.18082	$C_9H_{22}N_3^+$	0.23	z_3
186.19689	0.64	–			
215.11799	1.66	–			
229.13377	18.62	229.13354	$C_{14}H_{17}N_2O^+$	1.00	a_1
258.16048	1.06	–			
260.28134	5.73	260.28087	$C_{13}H_{34}N_5^+$	1.81	tz_4
272.17614	2.02	–			
286.19123	64.04	286.19139	$C_{17}H_{24}N_3O^+$	-0.56	a_2
303.21803	16.76	303.21793	$C_{17}H_{27}N_4O^+$	0.33	t_{a2}
325.23977	0.68	325.23867	$C_{20}H_{29}N_4^+$	3.38	b_3
343.24953	34.97	343.24924	$C_{20}H_{31}N_4O^+$	0.84	a_3
360.27607	1.54	360.27579	$C_{20}H_{34}N_5O^+$	0.78	t_{a3}
399.32389	1.61	–			
400.30750	5.57	400.30709	$C_{23}H_{38}N_5O^+$	1.02	a_4
417.33380	100.00	417.33364	$C_{23}H_{41}N_6O^+$	0.38	$[M+H]^+$

* this fragment ion is not generated by FRIOC

Supplementary Figure 2: 4-OH-IndAc3(OH)335(NMe3)+ (*Agelenopsis aperta*)

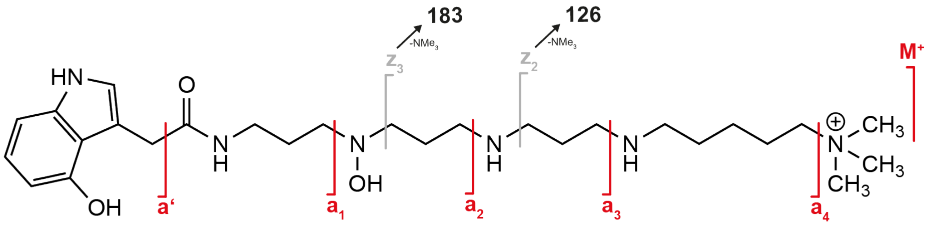

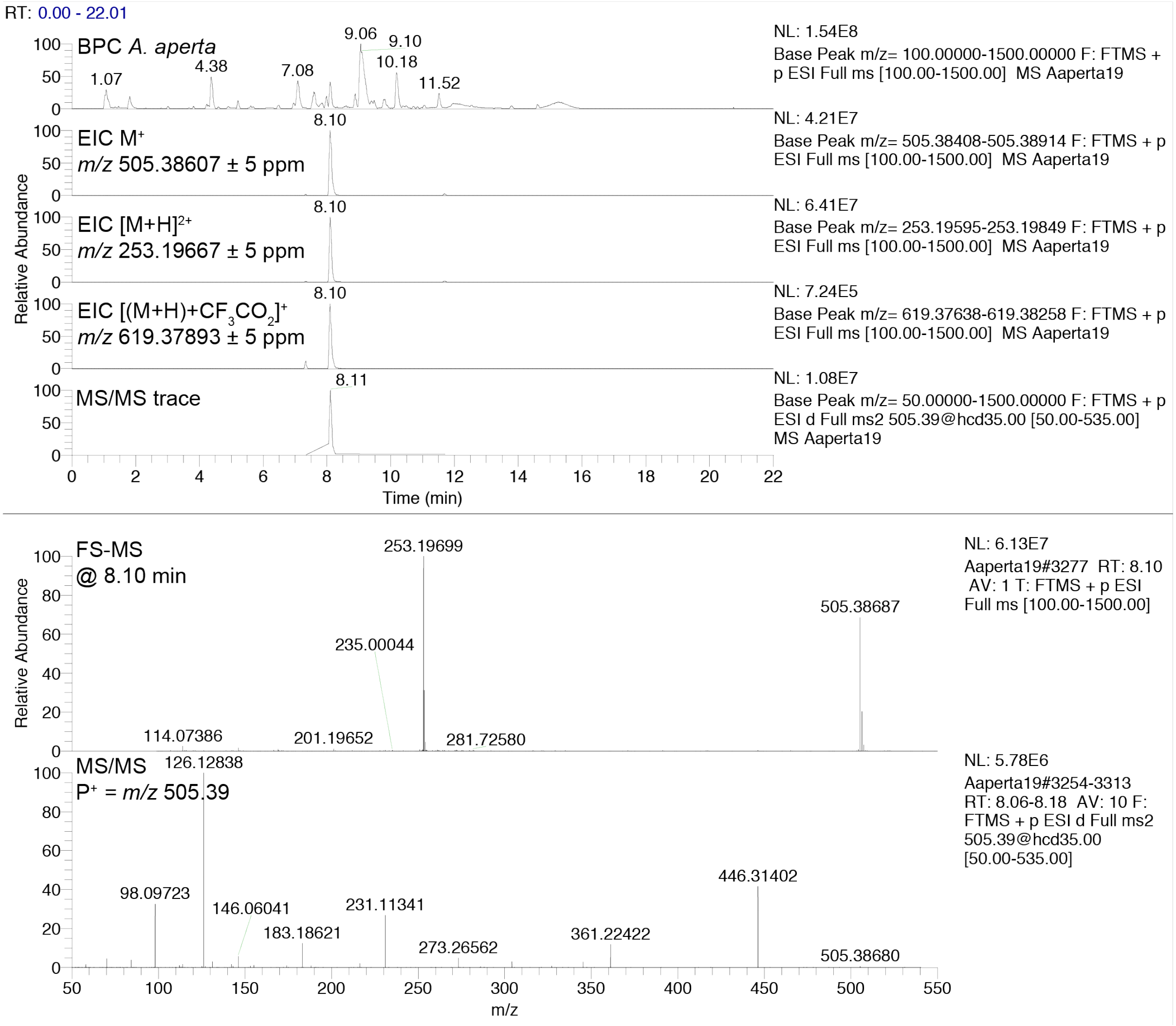

HDX
BPC *A. aperta*

HDX
EIC $M(D_6)^+$
m/z 511.42373 ± 5 ppm

HDX
EIC $[M(D_6)+D]^{2+}$
m/z 256.71864 ± 5 ppm

HDX
EIC $[(M(D_6)+D)+CF_3CO_2]^+$
m/z 626.42287 ± 5 ppm

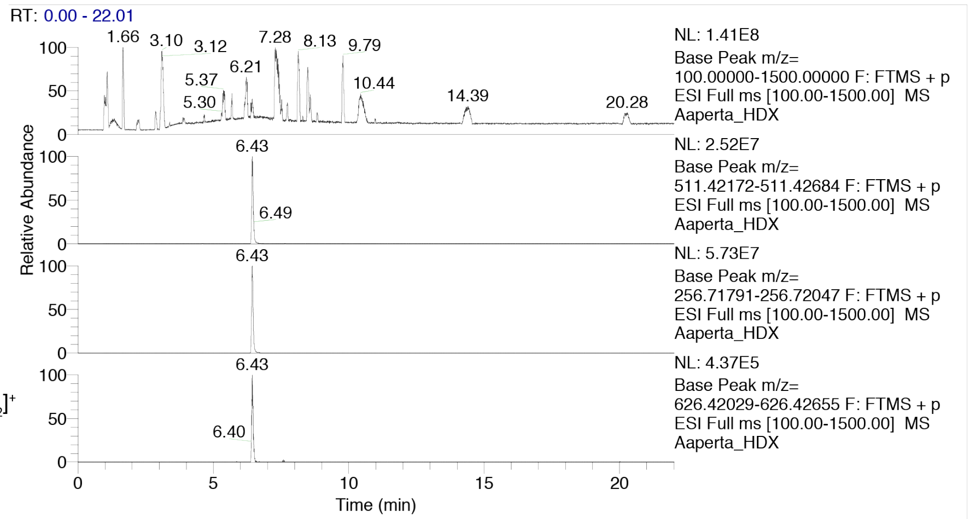

HDX
FS-MS
@ 6.43 min

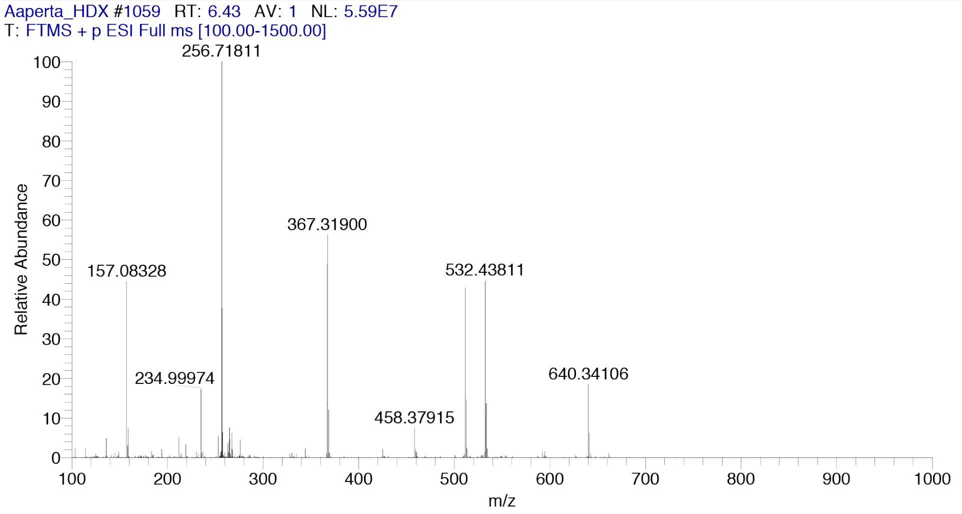

FS-MS / FS-MS HDX

	m/z detected	R_t [min]	*m/z* FRIOC	Molecular formula	Δ [ppm]
M^+	505.38687	8.10	505.38607	$C_{27}H_{49}N_6O_3^+$	1.58
$M(D_6)^+$	511.42300	6.43	511.42373	$C_{27}H_{43}D_6N_6O_3^+$	-1.43

MS/MS (P^+ = *m/z* 505.39)

m/z detected	Rel. Int.	*m/z* FRIOC	Molecular formula	Δ [ppm]	Fragment type
58.06612	1.51	–			
70.06610	4.55	–			
84.08169	3.83	–			
98.09723	32.66	–	$C_6H_{12}N^+$	8.16	y_2' *
112.11267	1.12	–			
114.09202	1.64	–			
125.12059	0.63	–			
126.12838	100.00	–	$C_8H_{16}N^+$	5.15	z_2 – NMe_3 *
127.12353	0.61	–			
131.11855	3.05	–			
142.12307	1.60	–			
143.15498	0.78	–			
146.06041	5.73	–	$C_9H_8NO^+$	2.53	a' *
153.13895	0.84	–			
155.15491	1.19	–			
174.05582	1.03	–	$C_{10}H_8NO_2^+$	5.00	a_0 *
183.18621	12.33	–	$C_{11}H_{23}N_2^+$	3.44	z_3 – NMe_3 *
188.17666	0.98	–			
216.20792	2.06	–	$C_{11}H_{26}N_3O^+$	4.02	t_{z3} – NMe_3 *
231.11341	27.40	231.11280	$C_{13}H_{15}N_2O_2^+$	2.64	a_1
273.26562	4.86	–	$C_{14}H_{33}N_4O^+$	2.67	t_{z4} – NMe_3 *
304.16639	3.14	304.16557	$C_{16}H_{22}N_3O_3^+$	2.70	a_2
327.21856	0.82	–			
345.22911	2.85	–			
361.22422	11.79	361.22342	$C_{19}H_{29}N_4O_3^+$	2.21	a_3
446.31402	42.64	446.31257	$C_{24}H_{40}N_5O_3^+$	3.25	a_4
505.38680	0.58	505.38606	$C_{27}H_{49}N_6O_3^+$	1.44	M^+

* this fragment ion is not generated by FRIOC

Supplementary Figure 3: 4-OH-IndAc3(OH)335(NMe₂) (*Agelenopsis aperta*)

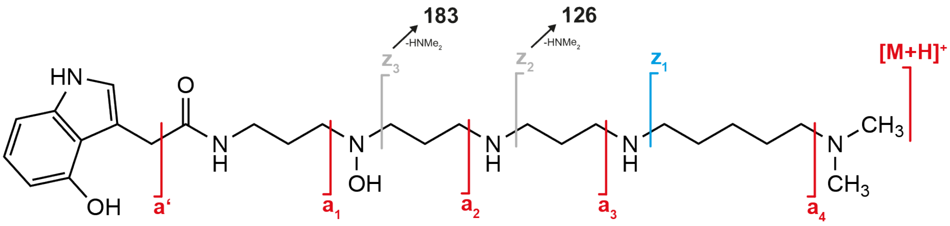

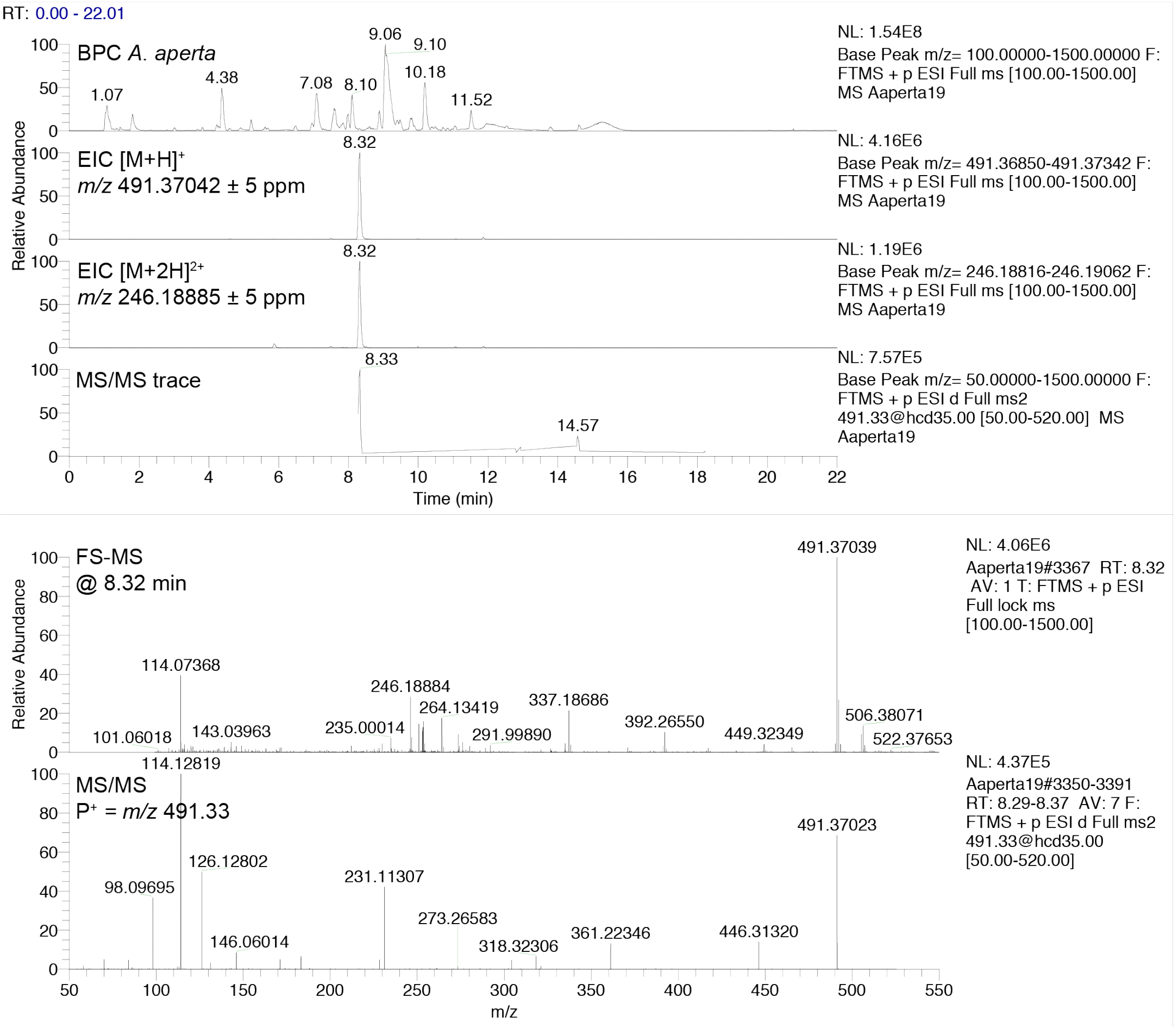

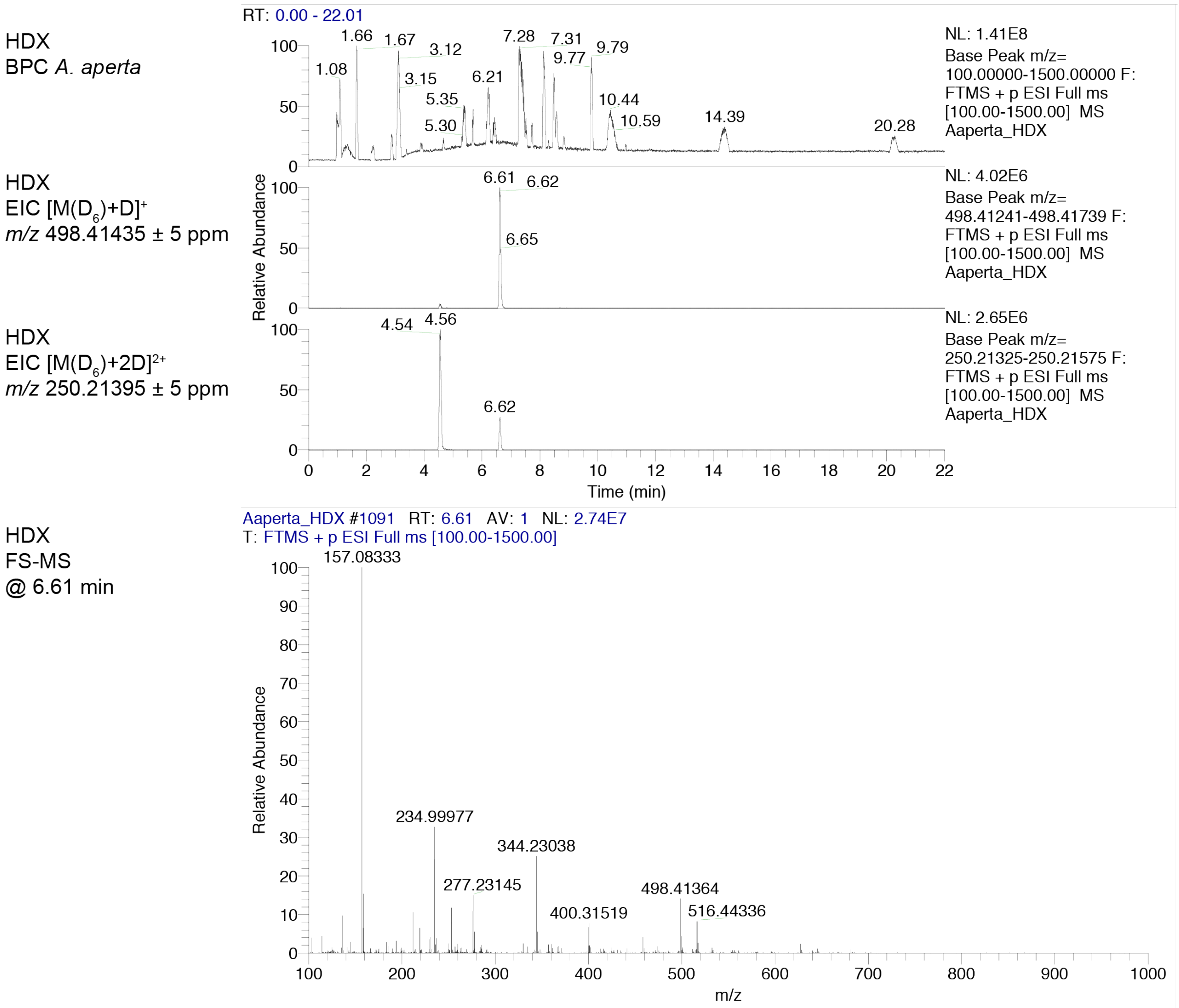

FS-MS / FS-MS HDX

	m/z detected	R_t [min]	*m/z* FRIOC	Molecular formula	Δ [ppm]
$[M+H]^+$	491.37039	8.32	491.37042	$C_{26}H_{47}N_6O_3^+$	-0.06
$[M(D_6)+D]^+$	498.41364	6.61	498.41435	$C_{26}H_{40}D_7N_6O_3^+$	-1.42

MS/MS (P^+ = *m/z* 491.37)

m/z detected	Rel. Int.	*m/z* FRIOC	Molecular formula	Δ [ppm]	Fragment type
58.06600	1.78	–			
70.06584	4.91	–			
84.08144	4.66	–			
86.09706	0.89	–			
98.09695	37.91	–	$C_6H_{12}N^+$	5.30	y_2' *
112.11271	1.31	–			
113.10795	1.12	–			
114.09179	2.65	–			
114.12819	100.00	114.12773	$C_7H_{16}N^+$	4.03	z_1
126.12802	49.78	–	$C_8H_{16}N^+$	2.30	z_2 – $NHMe_2$ *
131.11825	3.30	–			
143.15428	0.65	–			
146.06014	8.73	–	$C_9H_8NO^+$	0.68	a' *
171.18599	5.02	–			
183.18580	6.61	–	$C_{11}H_{23}N_2^+$	1.20	z_3 – $NHMe_2$ *
228.24420	4.76	–			
231.11307	42.42	231.11280	$C_{13}H_{15}N_2O_2^+$	1.17	a_1
231.13557	0.62	–			
261.26518	0.54	–			
273.26583	1.55	–	$C_{14}H_{33}N_4O^+$	3.44	t_{z4} – $NHMe_2$ *
304.16583	4.64	304.16557	$C_{16}H_{22}N_3O_3^+$	0.85	a_2
318.32306	6.61	–			
321.19306	1.50	–			
361.22346	13.13	361.22342	$C_{19}H_{29}N_4O_3^+$	0.11	a_3
446.31320	13.94	446.31257	$C_{24}H_{40}N_5O_3^+$	1.41	a_4
491.37023	68.84	491.37042	$C_{26}H_{47}N_6O_3^+$	-0.39	$[M+H]^+$

* this fragment ion is not generated by FRIOC
